# Accurate and efficient target prediction using a potency-sensitive influence-relevance voter

**DOI:** 10.1186/s13321-015-0110-6

**Published:** 2015-12-29

**Authors:** Alessandro Lusci, Michael Browning, David Fooshee, Joshua Swamidass, Pierre Baldi

**Affiliations:** School of Information and Computer Sciences, University of California, Irvine, Irvine, USA; Pathology and Immunology, Washington University in St. Louis, St. Louis, USA

**Keywords:** Target-prediction, Large-scale, Fingerprints, Molecular potency, Random inactive molecules, Influence-relevance voter

## Abstract

**Background:**

A number of algorithms have been proposed to predict the biological targets of diverse molecules. Some are structure-based, but the most common are ligand-based and use chemical fingerprints and the notion of chemical similarity. These methods tend to be computationally faster than others, making them particularly attractive tools as the amount of available data grows.

**Results:**

Using a ChEMBL-derived database covering 490,760 molecule-protein interactions and 3236 protein targets, we conduct a large-scale assessment of the performance of several target-prediction algorithms at predicting drug-target activity. We assess algorithm performance using three validation procedures: standard tenfold cross-validation, tenfold cross-validation in a simulated screen that includes random inactive molecules, and validation on an external test set composed of molecules not present in our database.

**Conclusions:**

We present two improvements over current practice. First, using a modified version of the influence-relevance voter (IRV), we show that using molecule potency data can improve target prediction. Second, we demonstrate that random inactive molecules added during training can boost the accuracy of several algorithms in realistic target-prediction experiments. Our potency-sensitive version of the IRV (PS-IRV) obtains the best results on large test sets in most of the experiments. Models and software are publicly accessible through the chemoinformatics portal at http://chemdb.ics.uci.edu/

**Electronic supplementary material:**

The online version of this article (doi:10.1186/s13321-015-0110-6) contains supplementary material, which is available to authorized users.

## Background

Several groups have proposed a wide range of algorithms capable of predicting the biomolecular targets of diverse molecules (see, for instance, Refs. [1–12]). These include protein structure-based methods [13, 14], and ligand-based methods such as pharmacophore searching [3], substructural analysis [15], and similarity searching [4]. Some methods, such as similarity searching using fingerprints [16, 17], are computationally faster than others, and can therefore be applied more efficiently to larger repositories of molecules [18, 19].

We can treat the virtual high-throughput screening (vHTS) task as a classification problem. Given a body of labeled training data—molecules known to be active or inactive—we want to classify untested molecules into one of those two groups. One approach is to represent each molecule by its fingerprint vector, placing it at some location in a high-dimensional space. We can then train a predictor (e.g., a neural network) which takes these fingerprint “coordinates” as inputs and decides on a class. This approach assumes the fingerprints contain information about the classification decision.

A second approach is to compute a similarity measure between each of the compounds, defining a “geometry” over the training molecules. Given a new molecule, we can compute N similarities of the molecule to those in the training set, and base our decision on these numbers. K-nearest neighbors (kNN) is a simple example of this approach. It looks at the *k* most similar neighbors and decides based on the majority class among them. This is a weak algorithm because it discards all other points outside of the *k*-neighborhood. Furthermore, it discards all of the N similarity values within that neighborhood. Contrast this with, say, a deep neural network that considers all N similarity values to make its decision. A slightly simpler version of such an approach would be an algorithm that looks at the N similarity values within the *k*-neighborhood, as is done by the influence relevance voter (IRV), an algorithm previously developed by our group [5]. The IRV is a shallow neural network that considers all of the similarities within the *k* closest neighbors to make its prediction. Incorporating this additional information about not only which neighbors a molecule is similar to, but how similar it is to each of them, allows the IRV to achieve state of the art results on benchmark data sets.

Various vHTS methods have predicted, and subsequent experiments have confirmed, drug-target interactions that were previously unknown. For example, Shoichet et al. [2] predicted thousands of unanticipated interactions by comparing 3665 FDA drugs against hundreds of targets. Thirty of these interactions were tested experimentally, and 23 new drug-target associations were confirmed. The methodology involved quantifying similarities as E values using the Similarity Ensemble Approach (SEA) [20] in order to build drug-target networks by linking drug-target interactions in accordance with the similarity values. Drugs were selected from the MDL Comprehensive Medicinal Chemistry database, while ligands were selected from the MDL Drug Data Report, WOMBAT [21], and StARlite databases. Molecules were represented as 2048-bit Daylight and 1024-bit folded ECFP-4 [20] topological fingerprints. Targets were represented as sets of ligands.

Similarly, Mestres et al. [22] used drug-target networks to model the relationships between diseases, genes, proteins, and molecules. They found that drugs targeting aminergic G protein-coupled receptors (GPRCs) showed the most promiscuous pharmacological profile. Molecules were described as sets of low-dimension descriptors called SHED [23]. Similarities were computed as euclidean distances.

Nadhi et al. [24] developed a model based on Bayesian statistics to allow the simultaneous evaluation of the biological effect of multiple compounds on multiple targets. Using data from WOMBAT, they reported 77 % accuracy for their predictions.

Meslamani et al. [7] presented an automated workflow to browse the target-ligand space. Their prediction system uses four ligand-based methods (SVM classification, SVR affinity prediction, nearest neighbors interpolation, and shape similarity) and two structure-based methods (docking and pharmacophore match). About 72 % of 189 clinical candidates were correctly identified by the proposed workflow. Ligand-based methods outperformed the accuracy of the structure-based ones, with no preference for any method in particular. The authors also showed that the quality of the predictions gradually increased with the number of compounds per target.

This work makes several contributions to the field. First, to the best of our knowledge, this is the first study that compares the performance of 5 well-established ligand-based methods to the recently introduced IRV. Second, this study not only confirms the findings of Meslamani et al. [7] regarding the relationship between number of ligands and prediction performance, but also brings deeper insight to the problem by demonstrating in greater detail how performance varies with the number of ligands. Third, this study introduces a potency-sensitive version of the IRV algorithm and shows that, in many cases, it is the best performing method among those tested, when the number of examples is large. This is an important result considering that the number of tested ligands per target in the ChEMBL dataset is expected to increase [25]. Fourth, we show performance improvements achieved by including random negatives during training. As an easily implemented strategy to boost the performance of SVM, RF, and IRV algorithms, this is also an important result.

## Methods

### Protein-molecule datasets

We use a dataset containing 490,760 molecule-protein interactions selected from the ChEMBL database [26] (version 13, February 2012), consisting of IC50 and EC50 values–the concentrations at which 50 % of target inhibition or activation is observed, respectively. As a measure of potency, we will refer to EC50 hereafter.

**Fig. 1:**
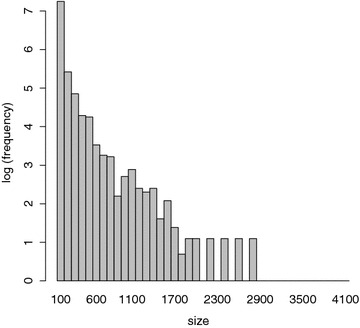
Distribution of the number of molecules associated with each protein. The *x* axis bins proteins by the number of small-molecule data-points with which they are associated. The *y* axis plots the number of proteins in each bin. There are 2108 protein targets. About 33 % of these datasets contain more than 100 molecules

This is similar data to that used in several other studies [6–12]. The data from PubChem is excluded because it often does not include EC50 potency data. Molecules were labeled inactive using three different cutoffs: 1, 5, and $10\,\upmu \hbox {M}$ concentrations. The entire dataset contains 3236 protein targets (cf. Additional file 1 containing the list of corresponding ChEMBL IDs). However, for 1128 of these protein targets, there are fewer than 10 active molecules. These were discarded for being too sparse to enable proper learning, but also because they cannot be used properly in the tenfold cross-validation experiments described below. That left 2108 protein targets with at least 10 molecules each. For 695 of these proteins, the corresponding datasets contain 100 molecules or more. The distribution of the dataset sizes associated with each protein is shown in Fig. 1.

There are several benchmark datasets available in the literature, but most of these datasets (1) do not contain potency data, (2) include data on only a small number of proteins, and (3) only contain closely-related molecules. ChEMBL is the most complete publicly available dataset for target prediction. It covers a large number of proteins and a large, diverse set of molecules, and a good portion of this data includes potency information. There are several errors in the ChEMBL data arising from both annotation mistakes and discrepancies in the literature. It is, however, a very common source of data for virtual screening. In particular, the highest quality data with the fewest discrepancies is the high potency actives data. There are commercial databases with similar data available but they require a licensing fee to access. For these reasons, ChEMBL is an ideal dataset on which to benchmark target prediction methods.

We extracted an external validation set from a newer version of ChEMBL (version 19, July 2014). The same protocol was used to extract all the new data-points added between version 13 and 19. These data-points were used as an independent set on which to test performance. The dataset consisted of 123,218 molecule-protein interactions, covering 66,707 different molecules, and 1016 protein targets.

In cases where multiple drug-target interactions were found, we used the average of the activities. We applied this protocol to the sets we used for both tenfold cross-validation and tenfold cross-validation with random negatives. However, we included multiple drug-target interactions in the external validation set.

### Activity and cutoffs

Each protein target (identified by its ChEMBL ID) in the dataset is associated with a certain number of molecules, together with the corresponding EC50 values expressed in $\upmu \hbox {M}$. A small EC50 value corresponds to high potency, i.e., only a small concentration of drug is required for EC50 bioactivity. A molecule is considered active against a certain target if its EC50 is lower than a certain cutoff value [27]. Unfortunately there is no agreement on which cutoff value should be chosen for a generic target-prediction problem, since the same cutoff could refer to different bioactivities in different assays. For example, a $10\,\upmu \hbox {M}$ cutoff could represent very active molecules in some assays, while also including only marginally active molecules [28]. Moreover, we wanted to ensure that our results were not overly dependent on a specific cutoff choice. For this reason, we decided to use three cutoff values: 1, 5 and $10\,\upmu \hbox {M}$. A molecule is labeled active if the corresponding EC50 is smaller than the selected cutoff, and inactive otherwise. As we will see, very similar results are observed across all cutoff values. In practice, the $1\,\upmu \hbox {M}$ cutoff may be most important because its data has the least noise.

### Random negative molecules

Active molecules are rare. Usually, less than one percent of molecules interact with a given protein. From the ChEMBL database, we selected a set of 10,000 molecules that do not bind any of the proteins in our study, and used them as random negatives during training as well as assessment. We refer to this set as the Random ChEMBL or “RC” dataset. RC was randomly split into two subsets: Train-RC including 1000 molecules, and Test-RC including the remaining 9000 molecules. Obviously this dataset can occasionally produce a false negative, however the advantages it provides in training and assessment outweigh the drawback of introducing a few false negatives. Note that some level of noise also exists among the positive molecules, due to inevitable variability in experimental methods.

### Molecular similarity

The more similar two molecules are, the more likely they are to have similar properties [29]. Fingerprint similarity is widely used in chemical informatics as a way of quantifying similarity between molecules [5, 30].

Fingerprints are vectors encoding the occurrence of certain substructures within a molecular graph. Each component of a fingerprint is a single bit which indicates either the presence (1-bit) or absence (0-bit) of a particular structure in the graph. We use a fingerprint very similar to the Extended Connectivity Fingerprint (ECFP) commonly used in the field. We use circular substructures [30, 31] of depth *d* up to 2 bonds, where atoms are labeled with their element and their connectivity (e.g., C3 for a carbon with three heavy atoms attached). Bonds are labeled according to their type (single, double, triple, or aromatic). Fingerprints tend to be very sparse and are easily compressible. In this paper, we used a lossless compression algorithm based on entropy encoding [32]. Similarity between fingerprints was measured using the Tanimoto metric [33].

The choice of fingerprint and its parameters affects the performance of all methods used in this study. However, the scope of this work does not include picking the optimal fingerprint. Instead it focuses on the machine learning component of target prediction. We control for the effect of fingerprints by using the exact same fingerprint across all methods.

### Mean similarity (MeanSim)

A commonly used and easily implemented way of classifying molecules is to score them by their average similarity to all known active molecules. This approach was extensively studied by Shoichet et al. [2] and ranks molecules identically to the method they ultimately propose. The Shoichet algorithm computes a new score, which orders molecules identically to MeanSim, and appropriately quantifies the statistical significance of each molecule-target association. The new score more accurately ranks targets associated with a test molecule than MeanSim. However, for a given target, it ranks collections of test molecules in the exact same order as MeanSim. Therefore, the performance of MeanSim in separating active and inactive molecules (the primary focus of this study) is exactly identical to the Shoichet algorithm.

### Maximum similarity (MaxSim)

Another commonly used and easily implemented way of classifying molecules based on known activities is Maximum Similarity. In this method, molecules are scored by their similarity to the most similar known active molecule. MaxSim is straightforward to implement, does not require any parameter tuning, has been well studied [34, 35], and is intuitively simple. The resulting predictions allow one to rank query molecules and examine the active molecule most similar to the query molecule, along with the corresponding similarity score, to gain some insights into the rationale behind a prediction.

In prior work, MaxSim has consistently outperformed MeanSim [36]. This is likely because MeanSim makes an implicit assumption that all the active molecules are in a single cluster in similarity space. MaxSim does not make this overly restrictive assumption, and thus can better capture cases where more than one class of molecules is active. Consequently, we expect MaxSim to outperform MeanSim.

### K nearest neighbors (kNN)

Another commonly used approach is *k* nearest neighbors. In contrast with MaxSim and MeanSim, kNN and the following methods use both active and inactive molecules to make predictions. Here, molecules are scored by the proportion of known actives amongst the *k* closest neighbors in the training set. For this study, we use $k = 11$, $k = 31$ and $k = 51$. Using these values we can investigate whether larger sets of neighbors lead to better performance.

### Support vector machines (SVM)

One of the most commonly used machine learning methods in virtual screening is Support Vector Machines (SVM) [37, 38]. SVMs are not easily implemented from scratch, but there are several good open source packages available. Part of their power comes from being able to use Tanimoto similarity between fingerprints explicitly [39]. SVMs frequently use the full training set of active and inactive molecules, and achieve nearly optimal performance. Our implementation of SVM uses the publicly available SVMTorch software [40]. The C and epsilon parameters were determined using the built-in parameter optimizer of the SVMTorch library that iterates over several possible values to pick the optimal choice.

### Random forest (RF)

A random forest [41] is an ensemble of decision trees, and is also commonly used in chemoinformatics to predict molecule activity. Given a training set {$${{\mathbf {x}}}_{{{\mathbf {i}}}}$$, $$y_{i}$$}, where $$y_{i}$$ is a molecular label (active, not active) and $${{\mathbf {x}}} \in {{\mathbb {R}}}^{D}$$ is a vector of features of length *D*, the first step consists of choosing a value for the parameter *m*, the number of tried attributes $$0<m < D$$, which is used to determine the splits at each node of each tree. Then *k* decision trees are grown using the training set and *k* random initialization seeds. The result is an ensemble of tree-structured classifiers {$$h({{\mathbf {x}}}_{{{\mathbf {i}}}}$$, $$y_{i})$$} where the output of the ensemble is the majority vote of the individual classifiers.

RFs have been applied in chemoinformatics to QSAR/QSPR modeling, and molecular classification problems [42–44]. Among the attractive features of the Random Forest approach are robustness and simplicity, including hyperparameter simplicity which corresponds essentially to choosing a single parameter (*m*). In practice, it has been shown that $m = \sqrt{D}$ is a good choice [45].

In this study, the input vector ${{\mathbf {x}}}_{{{\mathbf {i}}}}$ for each molecule *i*, is generated according to the following procedure: compute the Tanimoto similarity between *i* and each molecule *j* in the training set; sort the similarity values in descending order; take the first N values and multiply them by a binary activity coefficient $c_{j}$ defined as follows:

1 $$\begin{aligned} c_{j} = \left\{ \begin{array}{ll} 1 &{}\quad \text{ if }\,j\,\text{ is } \text{ active } \\ -1 &{} \quad \text{ if }\,j\,\text{ is } \text{ not } \text{ active } \\ \end{array}\right. \end{aligned}$$

After some exploration, we chose the following set of parameters: $D = 10$, $K = 200$, and $m =3 \approx \sqrt{D}$. Our implementation of Random Forests is based on the *sklearn.ensemble* library for Python [46].

### Influence-relevance voter (IRV)

The IRV was introduced by Swamidass et al. [5] and is not commonly used. However, it has several advantages over other methods. First, unlike RFs and SVMs, its predictions are easily interpretable in a manner similar to kNNs and MaxSim. Second, as we will see, it can be modified to take into account the potency of molecules in the training set. Third, it often outperforms SVM and RF methods.

Like kNN, the IRV looks at the neighborhood of *k* nearest neighbors. However it uses a neural network with shared weights to compute a more sophisticated function of this neighborhood, as opposed to the very simple majority membership used by kNN. Its output is defined as

2 $$\begin{aligned} z({{\mathcal {X}}}) = \sigma \left( w_{z} + \sum _{i=1}^{K}I_{i}\right) , \end{aligned}$$

where ${{\mathcal {X}}}$ is the test molecule, *i* ranges from 1 to *k* over all *k* nearest neighbors, $I_{i}$ is the “influence” of the $i \hbox {th}$ neighbor on the output, $w_{z}$ is the bias of the output node, and $\sigma (x)= 1/(1 + e^{-x})$ is the logistic function. These influences indicate exactly how much, and in which direction, each training example contributes to the predictions. The influence of the $i \hbox {th}$ node is defined by

3 $$\begin{aligned} I_{i} = R_{i}V_{i} \end{aligned}$$

where $R_{i}$ is the relevance and $V_{i}$ is the vote of the $i \hbox {th}$ neighbor. The relevance is defined as

4 $$\begin{aligned} R_{i} = \tanh (w_{y} + w_{s}s_{i} + w_{r}r_{i}) \end{aligned}$$

where $s_{i}$ is the similarity $S({{\mathcal {X}}}, N_i)$ of the $i \hbox {th}$ closest neighbor to the test molecule, $r_{i}$ is the rank of the $i \hbox {th}$ neighbor in the similarity-sorted list of neighbors, $w_{s}$ and $w_{r}$ are parameters tuning the importance of different inputs, and $w_{y}$ is the bias of the logistic unit.

The vote is defined by

5 $$\begin{aligned} V_{i} = \left\{ \begin{array}{ll} w_{0} &{} \quad \text{ if } c_{i} = 0 \\ w_{1} &{} \quad \text{ if } c_{i} = 1 \\ \end{array}\right. \end{aligned}$$

where $w_{0}$ is the weight associated with inactive neighbors, $w_{1}$ is the weight associated with active neighbors, and $c_{i} \in$ 0,1 is the class of the $i \hbox {th}$ neighbor.

The logistic output of the IRV can be interpreted as a probability and directly encodes the confidence of each prediction [47, 48]

$z({{\mathcal {X}}}) \approx ( {{\mathcal {X}}}$ is active — ${{\mathcal {X}}}$’s structure, training data)

In other words, the output of the network on a test molecule is approximately equal to the probability of the test molecule being active given its structure and the training data. This is enforced by training the network to minimize the relative-entropy or Kullback-Leibler divergence between the true target distribution $t({{\mathcal {T}}} )$ and the predicted distribution $z({{\mathcal {T}}} )$ across all molecules ${{\mathcal {T}}}$ in the training set. The IRV is trained by gradient descent to minimize the error, equivalently, the negative log-likelihood given by [47]

6 $$\begin{aligned} - \sum t({{\mathcal {T}}})\log \left[ z({{\mathcal {T}}})\right] + (1-t({{\mathcal {T}}}))\log \left[ 1-z({{\mathcal {T}}})\right] , \end{aligned}$$

where the summation is over the training instances, ${{\mathcal {T}}}$. The IRV can be fine-tuned with several variations. In this study, setting the number of neighbors to 6 yielded a good compromise between total training time and accuracy of predictions.

### Potency-sensitive influence relevance voter (PS-IRV)

The IRV as we have defined it, along with most other machine learning approaches to target-prediction, completely ignores the potency of active and inactive molecules. However one may expect that potency is important to prediction and may contain useful information. Thus we also design a version of the IRV that is sensitive to potency, the Potency-Sensitive IRV (PS-IRV).

In this study, we use three different cutoff values to assign the class $c_{i}$ to a molecule *i* . If the activity of *i* (i.e., its EC50) is less than the cutoff, $c_{i} = 1$ ($c_{i} = 0$, otherwise). In Eq. 5, we define the vote $V_{i}$ as a function of the class $c_{i}$. Therefore, $V_{i}$ depends indirectly on the activity of the molecule. It would be interesting to change Eq. 5 such that the vote depends directly on the activity of the molecule. There are obviously many ways to do this. Here we associate a weight to each cutoff, so that the vote is defined by

7 $$\begin{aligned} V_{i} = \left\{ \begin{array}{ll} w_{0} &{} \quad \text{ if } \;a_{i} < 1\,\upmu M \\ w_{1} &{} \quad \text{ if } \;a_{i} < 5\,\upmu M \\ w_{2} &{} \quad \text{ if } \;a_{i} < 10\,\upmu M \\ w_{3} &{} \quad \text{ if } \;a_{i} \ge 10\,\upmu M \\ \end{array}\right. \end{aligned}$$

We expect that in some cases this approach could outperform the standard IRV method, because its input includes the potencies of the neighbors.

In this formulation the uncertainty associated with each datapoint is not used by the model. However, uncertainty could be added in many ways. For example, the vote could be set to the average votes (computed using this formula) of all observed EC50s of a neighbor. In this way, the uncertainty inherent in conflicting measurements would be directly encoded in the IRV votes. We expect there could be performance gains from using this approach and others like it, but we leave that to future work.

### Performance metrics

Performance of different target-prediction methods is quantified using two standard metrics: the area under the ROC curve (AUC) [49], and the enrichment of the prediction ranked test data [50]. The ROC curve plots the fraction of correctly predicted actives, i.e. the true positive rate (TPR), versus the fraction of inactives incorrectly predicted as actives, i.e. the false positive rate (FPR). We calculate this for each chosen threshold. The enrichment metric gives the percentage of true actives found at the top of the ranked list of predictions. In the results, we use four different percentages to define the top list: 5, 10, 20, and 30 %. Whereas AUC quantifies the overall accuracy of separation between actives and inactives, enrichment rank quantifies the ability of a method to identify actives within specific top *N*  % cutoffs.

## Results

In the following sections we present the performance of these approaches on the ChEMBL dataset. We first present the results obtained using a standard cross-validation approach. Second, we show similar performance using a simulated target-prediction experiment where negative molecules are used during the assessment. Third, we find that using random negatives during training improves the performance of the machine learning approaches in the simulated target-prediction experiments. Finally, confirming prior work, we show that the IRV yields readily interpretable models. The trends we observe in the incremental plots are consistent across all cutoffs, therefore we only include the figures referring to $1\,\upmu \hbox {M}$ cutoff. The full list of figures is available in Additional file 2.

### Cross-validation assessment

The computational models were first trained and tested using tenfold cross-validation. Each set of training molecules was randomly divided into 10 equally sized sets or folds. Each fold was used for testing once, while the remaining nine folds were combined into a training set. This was repeated ten times, the outputs of each fold were combined, and the performance assessed. The entire procedure was then repeated over each target whose corresponding set of molecules contained at least 10 molecules, corresponding to 2108 proteins. Note that there are some minor variations in the actual number of proteins used in some of the tests. For instance, it does not make sense to apply 31NN to a training set with 31 molecules or fewer, as the output would be constant.

**Fig. 2:**
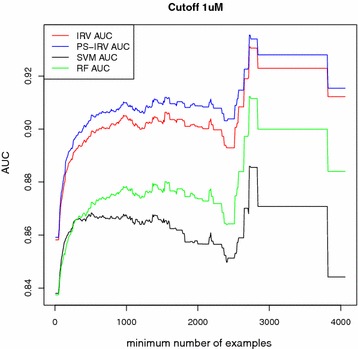
Cross-validation experiment: AUC scores as dataset size grows. Average AUC (*y* axis) plotted as a function of the minimum number of training molecules on the *x* axis. Model performance (AUC) increases as datasets with fewer examples are excluded

**Fig. 3:**
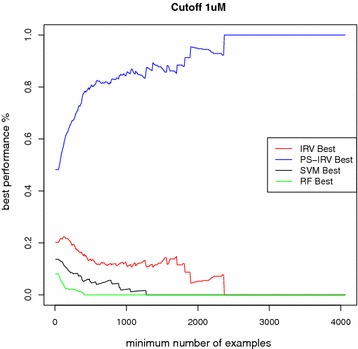
Cross-validation experiment: best performing models as dataset size grows. The fraction of times each model achieves the best performance for a dataset is plotted on the *vertical axis*, excluding datasets containing a number of molecules smaller than a specified size. PS-IRV is more consistently the best performer as more of the smaller datasets are excluded

We discarded all targets that did not meet the minimum requirement of having at least one example of both classes in each fold. Average performances are reported here: (Table 1; Figs. 2, 3). For brevity, only the best performing nearest neighbor method, 11NN, is included, since we observed that performance did not improve for greater values of *k* . Likewise, for brevity, we only show AUC performance. In Additional files (cf. Additional file 2: Tables S1, S3), we also report enrichment at several cutoff values (5, 10, 20, and 30 %), as well as the results corresponding to 31NN and 51NN.

We note several trends. First, performance is related to dataset size. Proteins with the largest number of known ligands yield the best performance, and excluding the smallest datasets increases average performance. Second, machine learning methods, which tune their parameters to the data, (SVM, RF, IRV, and PS-IRV) on average outperform the methods that are not tuned to the data (MaxSim, MeanSim, and 11NN), and have similar performances amongst each other. The performance disparity between machine learning methods and the other methods was strongest for the largest datasets. Third, PS-IRV becomes the best performing method as the number of examples in the training set increases. Fourth, the MeanSim method is consistently the worst performing method. All four trends are robust and observed across all cutoffs and both assessment methods.

Many of the differences underlying these trends are small, but they are statistically significant. A paired t test of the AUC values (where AUC performances are paired by target) shows almost all of these average AUC differences to be significant at a conservative 0.005 p value cutoff (cf. Additional file 2: Tables S4, S5).

One of the limitations of this assessment is that cross-validation estimates the prediction error on new test molecules as if these were drawn from a distribution over chemical space similar to that of the training set. Often the training sets used are small and potentially biased, containing, for instance, more active than inactive examples. For our data, the average percentage of positive examples was close to 50: 41, 57 and 63 %, for each of the three EC50 cutoffs respectively, so this is not a major concern.

We further assessed the machine learning methods PS-IRV, SVM, and RF, by measuring their performance at classifying external data obtained from a later version of ChEMBL (Table 2). For brevity, we only present the average AUC, but enrichment results are available in the Additional files section (cf. Additional file 2: Table S3). Overall, the methods obtain similar results, although PS-IRV slightly outperforms the other methods. A paired t test of the AUC values (where AUC performance is paired by target) is available in Additional files (cf. Additional file 2: Tables S6, S7). The results show a performance drop in comparison to the results of the tenfold cross-validation experiments. This drop is reasonable because the tested approaches, as they are based on fingerprint similarity, will fail at predicting active molecules that are not similar to known actives. This is a well known point of failure in similarity based approaches.

### Accuracy in simulated target-prediction

**Fig. 4:**
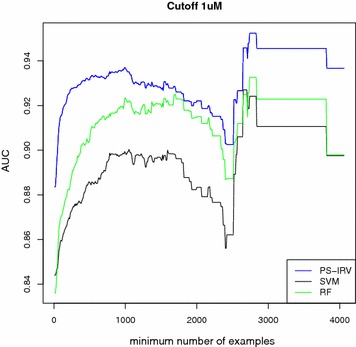
Simulated target-prediction experiment: AUC scores as dataset size grows. Average AUC (*y* axis) plotted as a function of the minimum number of training molecules (*x* axis). Each method’s ability to separate known actives from a background set of 9000 random ChEMBL molecules, assumed to be inactive, is measured. Training sets are not augmented

We address the inherent limitation to the use of cross-validation by simulating a more realistic target-prediction experiment as a proxy. To do so, we use the same trained models from the tenfold cross-validation procedure, but augment their test sets with a background dataset of 9000 molecules drawn at random from ChEMBL (the Test-RC dataset). Here we report the results only for the SVM, RF, and PS-IRV models (Table 3; Fig. 4). For brevity, we only report the AUC results, but enrichment values are available in Additional file (cf. Additional file 2: Table S8).

The simulated target-prediction results are on average similar to the corresponding cross-validation results. Model performances do not drop significantly, which is a sign of robustness. There are however some subtle differences between simulated target-prediction and cross-validation. A paired t test of the AUC values (where AUC performances are paired by target) is available in Additional files (cf. Additional file 2: Tables S9, S10). For instance, the PS-IRV performs better for a $1\,\upmu \hbox {M}$ cutoff and worse for 5 and $10\,\upmu \hbox {M}$ cutoffs. RF shows a similar trend, but with a more significant degradation in performance. In contrast, SVM shows only slightly worse results for $1\,\upmu \hbox {M}$, and better ones for 5 and $10\,\upmu \hbox {M}$. Most importantly, PS-IRV has been confirmed to outperform the other methods on large sets at every cutoff. These same trends are observed when assessing methods by enrichment.

### Training with random negatives

**Fig. 5:**
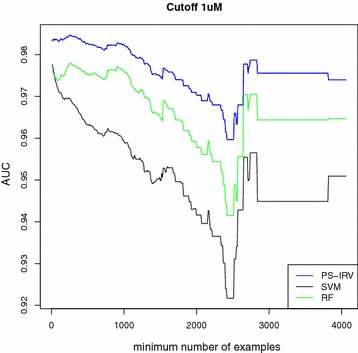
Simulated target-prediction experiment when training with random negatives: AUC scores as dataset size grows. Average AUC (*y* axis) plotted as a function of the minimum number of training molecules (*x* axis). Each method’s ability to separate known actives from a background set of 9000 random ChEMBL molecules, assumed to be inactive, is measured. 1000 random negative molecules are added to the original training sets. The extended training sets result in significant performance improvements

In this section, we investigate an approach to further improve performance of the models in the simulated target-prediction assessment by adding a sample of random negative molecules to the training set, labeling them all inactive. Once again, we assess the trained models using the simulated target-prediction protocol described in the previous section. This approach proves to be quite effective (Tables 3, 4; Fig. 5), as both the AUC and the enrichment metrics are well above 0.90 and 90 %, respectively. This trend is observed across all cutoffs and dataset sizes, using both AUC and enrichment metrics. Finally, we assess the performance of the models trained with random negative molecules in a simulated target-prediction experiment, including the external data from a later version of ChEMBL (Table 5). For brevity, we only report the AUC results, but enrichment results are available in Additional files (cf. Additional file 2: Table S11). Overall, the models achieve excellent results for both AUC and enrichment metrics. We observe a small performance drop in comparison to the simulated-target prediction experiment that did not include external ligands. Furthermore, PS-IRV outperforms the other methods at the $1 \upmu \hbox {M}$ cutoff, and matches SVM at the 5 and $10\, \upmu \hbox {M}$ cutoffs. In contrast, RF shows slightly worse performance than the other methods. A paired T-test of the AUC values (where AUC performances are paired by target) is available in Additional files (cf. Additional file 2: Tables S12, S13).

### Predictions and interpretability

Target-prediction methods can be used to screen chemical libraries containing millions of molecules for potential bioactivity and medical relevance. Given a target, one can use these techniques to rapidly screen for molecules with predicted activity against the target. Another approach is to use target-prediction methods to predict the bioactivity of a single molecule against many possible targets. We generally refer to this problem as target “deconvolution” [28], which is used to identify novel targets for molecules that already have known target activity [2, 51]. All the methods presented here can be applied to both problems. One of the major issues with target “deconvolution” is model comparability, i.e., comparing models that have been trained independently [52]. It is common practice to train an independent model for each of several potential targets. Given a test molecule, each target’s model is used to obtain a prediction score, and these scores are sorted to rank the targets. This method assumes that the scores obtained from the different models are directly comparable, a requirement that is not necessarily satisfied unless one imposes some constraints on the output of the models [52]. In contrast, probabilistic predictions make direct comparison possible [28]. More specifically, the output *O* of a model is a well tuned probability if, for example, a value $O = 0.6$ means that approximately 60 % of the molecules with a score of 0.6 are true actives [53].

**Fig. 6:**
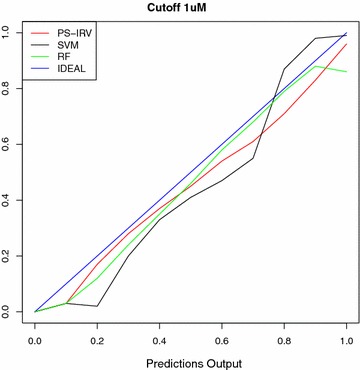
Probabilistic predictions. This reliability diagram plots the percentage of positive molecules (*y* axis) in the respective bins of molecules with similar prediction values (*x* axis). The data is collected from the outputs of the target-prediction models with AUC greater than 0.90. The PS-IRV and RF both produce lines that closely follow the *y* = *x* *line*, indicating that their output can be interpreted as a probability

Here we study this condition for the main machine learning methods included in this study: SVM, RF, and PS-IRV. To make sure that the learning process was successful for each model, we select only the targets for which each classifier scored an AUC greater than 0.90. We then sort the predictions in ascending order and partition them into bins of size 0.1. Then we compute the percentage of active molecules for each bin. We iterate this procedure over each of the methods for each cutoff. For SVM and RF, we scale the values to be within the range (0.0, 1.0). The results are shown in Fig. 6. For SVM, we see that the curve deviates from the ideal straight line. This is reasonable because the output of an SVM model is not directly interpretable as a probability. The curves for both PS-IRV and RF are fairly linear. However, we notice that RF fails to assign the correct probability to the outputs between 0.9 and 1.0.

**Fig. 7:**
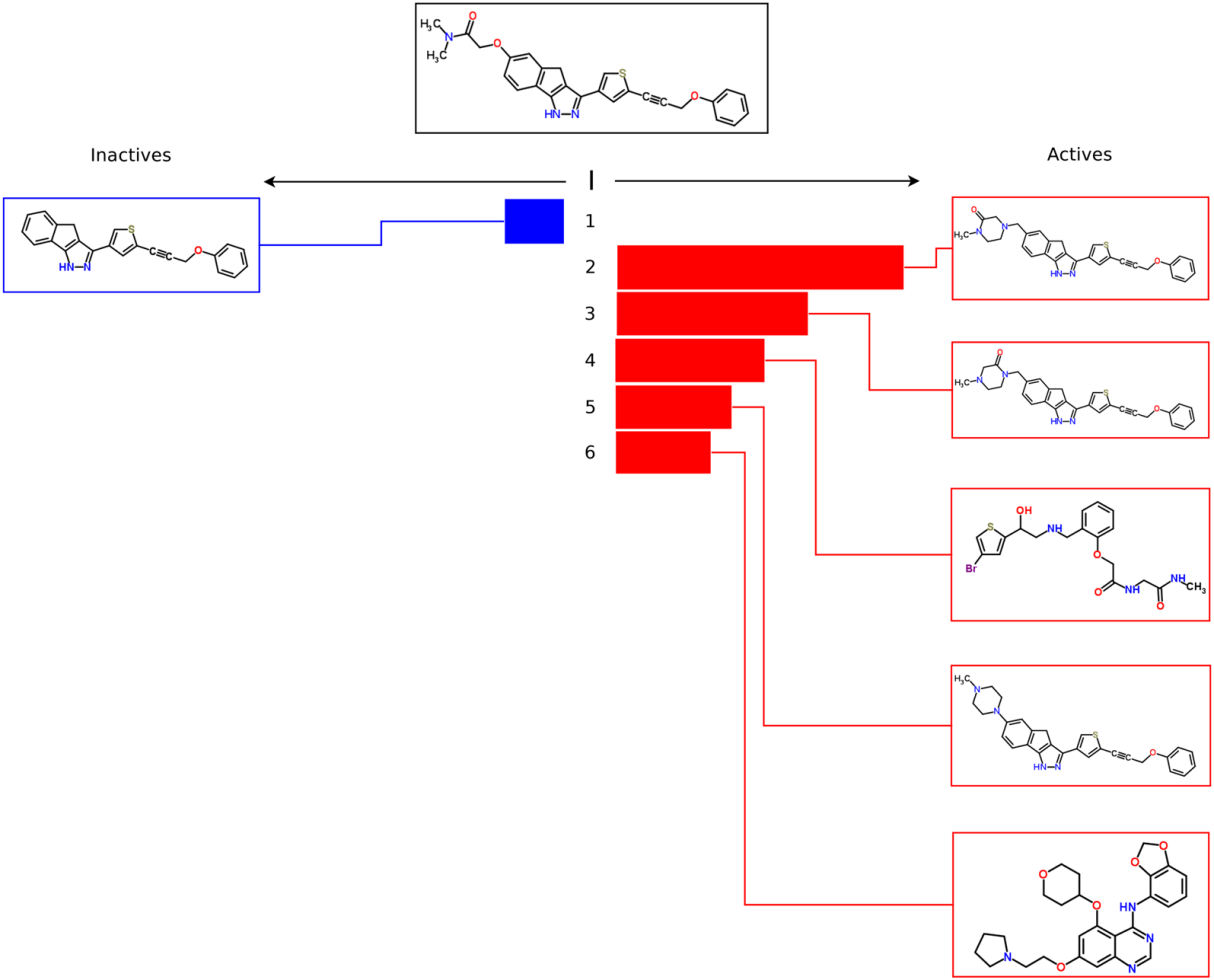
IRV Interpretability. A test molecule is shown at center, along with its neighbors and their influences. Each neighbor’s influence factors into the overall vote determining the predicted activity of the test molecule. This test molecule has been experimentally determined as active, and is predicted by IRV to be active given its neighbors and their influences

Machine learning methods can produce good results in target prediction and other domains [54–56], but they are sometimes criticized as being “black-box” methods, and therefore difficult to interpret. One of the advantages of the IRV over other machine learning methods is precisely its interpretability. Specifically, it exposes the data used to classify each test molecule in such a way that it can be readily visualized and understood. We demonstrate this in Fig. 7 by showing an example taken from the tenfold cross-validation data and how the influence of the neighbors can be visualized. The molecule on the left side is inactive and the molecules on the right side are active (see the original IRV paper [5] for additional details).

### Target-prediction web service

For more than 1500 proteins, the PS-IRV models achieve greater than 0.9 AUC performance in simulated target-prediction experiments. These models are available through a web server on our chemoinformatics portal (http://chemdb.ics.uci.edu/). Users may submit up to 50 molecules in SMILES format, and the request is processed offline. Once the computation is completed, the server sends the results file to a user-specified email address. Results are shown in three comma-separated value tables, each one corresponding to a different cutoff value. Independent tables are generated for each input molecule. In each table, the predicted activities of an input molecule are sorted in ascending order. The targets are identified by their corresponding ChEMBL IDs and preferred names. More information can be found at the chemoinformatics portal at http://chemdb.ics.uci.edu/.

## Conclusion

In this study we conducted a large-scale assessment of several target-prediction methods using a large dataset of molecule-protein target pairs selected from the ChEMBL database. Methods were compared along several dimensions, by computing the corresponding average AUC and enrichment rank over all the targets. As expected, the more sophisticated machine learning methods outperformed simpler approaches like MaxSim, MeanSim, and kNN. IRV-based methods compared favorably with SVMs and RFs. Finally, we introduced a variant of the basic IRV method, the Potency-Sensitive IRV, which showed a small but statistically significant performance improvement over other methods by leveraging potency information. We also demonstrated that adding random negative molecules to the training sets dramatically improved the ability of the PS-IRV, SVM, and RF models to identify active molecules from a large set of inactive ones. Finally, we showed how IRV-based methods have the advantage of producing a probabilistic output which is easily interpreted visually. We leave for future work the application of even more complex methods, such as undirected graph recursive neural networks (UG-RNNs) [57], to large-scale drug-target screening problems.

**Table 1:** AUC performance in the cross-validation experiment on the ChEMBL dataset

Cutoff ($\upmu \hbox {M}$)	MaxSim	MeanSim	11NN	IRV	PS-IRV	SVM	RF
All datasets							
$1$	0.79	0.76	0.81	*0*.*86*	*0*.*86*	0.84	0.84
$5$	0.76	0.74	0.82	0.84	*0*.*85*	0.84	0.82
$10$	0.75	0.73	0.81	*0*.*84*	*0*.*85*	0.84	0.82
Datasets with fewer than 100 molecules							
$1$	0.75	0.75	0.75	*0*.*78*	*0*.*78*	0.77	*0*.*78*
$5$	0.72	0.73	0.74	0.74	0.76	*0*.*77*	0.76
$10$	0.71	0.71	0.74	0.75	0.75	*0*.*76*	*0*.*76*
Datasets with more than 100 molecules							
$1$	0.80	0.76	0.73	0.87	*0*.*88*	0.85	0.85
$5$	0.77	0.74	0.84	0.87	*0*.*88*	0.86	0.84
$10$	0.77	0.73	0.84	0.87	*0*.*88*	0.86	0.86
Datasets with more than 200 molecules							
$1$	0.81	0.75	0.84	0.89	*0*.*89*	0.86	0.86
$5$	0.78	0.74	0.86	0.89	*0*.*90*	0.87	0.86
$10$	0.77	0.73	0.86	0.88	*0*.*90*	0.87	0.85

Each section of the table shows the average performance for datasets of different sizes

Best results within each group are in italics

**Table 2:** AUC performance in the cross-validation experiment on the external validation (ChEMBL 19) dataset

Cutoff ($\upmu \hbox {M}$)	PS-IRV	SVM	RF
All datasets			
$1$	*0*.*70*	0.69	0.68
$5$	*0*.*69*	0.67	0.67
$10$	*0*.*69*	0.66	0.67
Datasets with more than 100 molecules			
$1$	*0*.*71*	0.70	0.70
$5$	*0*.*70*	0.68	0.69
$10$	*0*.*70*	0.67	0.67
Datasets with more than 200 molecules			
$1$	*0*.*72*	*0*.*72*	0.71
$5$	*0*.*71*	0.69	0.70
$10$	*0*.*70*	0.68	0.68

Models were trained on the ChEMBL 13 dataset

Each section of the table shows the average performance for datasets of different sizes

Best results within each group are in italics

**Table 3:** AUC performance in the simulated target-prediction experiments

Method	Average AUC ($1\,\upmu \hbox {M}$)	Average AUC ($5\,\upmu \hbox {M}$)	Average AUC ($10\,\upmu \hbox {M}$)
Training without random negatives			
PS-IRV	*0*.*88*	0.84	0.83
SVM	0.84	*0*.*85*	*0*.*85*
RF	0.84	0.80	0.79
Training with random negatives			
PS-IRV	*0*.*98*	*0*.*98*	0.97
SVM	*0*.*98*	*0*.*98*	0.98
RF	*0*.*98*	*0*.*98*	*0*.*98*

Models were trained using a tenfold cross-validation protocol and tested on the corresponding test set augmented with 9000 randomly selected ChEMBL molecules

In the top panel, models were trained in the standard way, without random negatives. In the bottom panel, the training set was supplemented with 1000 random negatives

Adding random negatives dramatically improves the performance of all methods

Best results are in italics

**Table 4:** Average enrichment in the simulated target-prediction experiment when training with random negatives

Enrichment (%)	PS-IRV	SVM	RF
$1\,\upmu \hbox {M}$ cutoff			
5	*96*	92	95
10	*98*	94	96
20	*98*	97	97
30	*99*	*98*	97
$5\,\upmu \hbox {M}$ cutoff			
5	*95*	92	*95*
10	*97*	94	96
20	*98*	97	97
30	98	98	97
$10\,\upmu \hbox {M}$ cutoff			
5	*94*	*94*	93
10	*96*	95	*96*
20	*97*	*97*	*97*
30	97	*98*	97

Models are tested using 10-fold cross-validation. 9000 randomly selected ChEMBL molecules are added to the original test set as putative inactives. 1000 randomly selected ChEMBL molecules are added to the original training sets as putative inactives. Best results at each cutoff are in italics

**Table 5:** AUC performance in the simulated target-prediction experiment including external validation molecules

Method	$1\,\upmu \hbox {M}$	$5\,\upmu \hbox {M}$	$10\,\upmu \hbox {M}$
Average AUC			
PS-IRV	*0*.*90*	*0*.*87*	*0*.*86*
SVM	0.88	0.86	*0*.*86*
RF	0.85	0.84	0.84
Median AUC			
PS-IRV	*0*.*96*	*0*.*94*	*0*.*93*
SVM	0.94	0.93	*0*.*93*
RF	0.93	0.91	0.90

Models are trained using 10-fold cross-validation and tested on the external validation set

Training and test sets are augmented with 1000 and 9000 random negative molecules respectively

Here, we report both average and median AUC as we find a significant difference between the two measures. The results suggest that if we exclude a few outliers, AUC performance is consistently above 0.90 for each method. Best results are in italics

## Additional files

10.1186/s13321-015-0110-6 Protein targets list.
10.1186/s13321-015-0110-6 Additional tables and figures.

## References

[1] Lounkine E, Keiser MJ, Whitebread S, Mikhailov D, Hamon J, Jenkins JL, Lavan P, Weber E, Doak AK, Cote S (2012). Large-scale prediction and testing of drug activity on side-effect targets. Nature.

[2] Keiser MJ, Setola V, Irwin JJ, Laggner C, Abbas AI, Hufeisen SJ, Jensen NH, Kuijer MB, Matos RC, Tran TB (2009). Predicting new molecular targets for known drugs. Nature.

[3] Schwab CH (2011). Conformations and 3d pharmacophore searching. Drug Discov Today Technol.

[4] Ripphausen P, Nisius B, Bajorath J (2011). State-of-the-art in ligand-based virtual screening. Drug Discov Today.

[5] Swamidass SJ, Azencott C-A, Lin T-W, Gramajo H, Tsai S-C, Baldi P (2009). Influence relevance voting: an accurate and interpretable virtual high throughput screening method. J Chem Inf Model.

[6] Simon Z, Peragovics Á, Vigh-Smeller M, Csukly G, Tombor L, Yang Z, Zahoránszky-Kőhalmi G, Végner L, Jelinek B, Hári P (2011). Drug effect prediction by polypharmacology-based interaction profiling. J Chem Inf Model.

[7] Meslamani J, Bhajun R, Martz F, Rognan D (2013). Computational profiling of bioactive compounds using a target-dependent composite workflow. J Chem Inf Model.

[8] Heikamp K, Bajorath J (2011) Large-scale similarity search profiling of chembl compound data sets. J Chem Inf Model 51(8):1831–1839 10.1021/ci200199u 21728295

[9] Vidal D, Mestres J (2010) In silico receptorome screening of antipsychotic drugs. Mol Inf 29(6-7):543–551 10.1002/minf.201000055 27463332

[10] Sugaya N (2013) Training based on ligand efficiency improves prediction of bioactivities of ligands and drug target proteins in a machine learning approach. J Chem Inf Model 53(10):2525–2537 10.1021/ci400240u 24020509

[11] Sugaya N (2014) Ligand efficiency-based support vector regression models for predicting bioactivities of ligands to drug target proteins. J Chem Inf Model 54(10):2751–2763 10.1021/ci5003262 25220713

[12] Alvarsson J, Eklund M, Engkvist O, Spjuth O, Carlsson L, Wikberg JE, Noeske T (2014) Ligand-based target prediction with signature fingerprints. J Chem Inf Model 54(10):2647–2653 10.1021/ci500361u 25230336

[13] Simmons KJ, Chopra I, Fishwick CW (2010). Structure-based discovery of antibacterial drugs. Nat Rev Microbiol.

[14] Lill M (2013) Virtual screening in drug design. In: In Silico Models for Drug Discovery. Springer, New york, pp. 1–12

[15] Breault GA, Comita-Prevoir J, Eyermann CJ, Geng B, Petrichko R, Doig P, Gorseth E, Noonan B (2008). Exploring 8-benzyl pteridine-6, 7-diones as inhibitors of glutamate racemase (muri) in gram-positive bacteria. Bioorg Med Chem Lett.

[16] Baldi P, Nasr R (2010) When is chemical similarity significant? the statistical distribution of chemical similarity scores and its extreme values. J Chem Inf Model:1205–1222 **(in press)** 10.1021/ci100010v PMC2914517 20540577

[17] Nasr R, Vernica R, Li C, Baldi P (2012). Speeding up chemical searches using the inverted index: the convergence of chemoinformatics and text search methods. J Chem Inf Model.

[18] Chen J, Swamidass SJ, Dou Y, Bruand J, Baldi P (2005) ChemDB: a public database of small molecules and related chemoinformatics resources. Bioinformatics 21:4133–4139 10.1093/bioinformatics/bti683 16174682

[19] Chen JH, Linstead E, Swamidass SJ, Wang D, Baldi P (2007). ChemDB update-full-text search and virtual chemical space. Bioinformatics.

[20] Hert J, Keiser MJ, Irwin JJ, Oprea TI, Shoichet BK (2008). Quantifying the relationships among drug classes. J Chem Inf Model.

[21] Olah M, Mracec M, Ostopovici L, Rad R, Bora A, Hadaruga N, Olah I, Banda M, Simon Z (2004) Wombat: world of molecular bioactivity. Chemoinformatics Drug Discov 1

[22] Gregori-Puigjané E, Mestres J (2008). A ligand-based approach to mining the chemogenomic space of drugs. Comb Chem High Throughput Screen.

[23] Mestres J, Gregori-Puigjané E, Valverde S, Solé RV (2009). The topology of drug-target interaction networks: implicit dependence on drug properties and target families. Mol Biosyst.

[24] Nidhi GM, Davies JW, Jenkins JL (2006). Prediction of biological targets for compounds using multiple-category bayesian models trained on chemogenomics databases. J Chem Inf Model.

[25] Heikamp K, Bajorath J (2013). The future of virtual compound screening. Chem Biol Drug Des.

[26] ChEMBL (2014)

[27] Hausmann H, Richters A, Kreienkamp HJ, Meyerhof W, Mattes H, Lederis K, Zwiers H, Richter D (1996). Mutational analysis and molecular modeling of the nonapeptide hormone binding domains of the [arg8]vasotocin receptor. Proc Natl Acad Sci USA.

[28] Koutsoukas A, Lowe R, KalantarMotamedi Y, Mussa HY, Klaffke W, Mitchell JB, Glen RC, Bender A (2013). In silico target predictions: defining a benchmarking data set and comparison of performance of the multiclass naive bayes and parzen-rosenblatt window. J Chem Inf Model.

[29] Johnson MA, Maggiora GM (1990). Concepts and applications of molecular similarity.

[30] Rogers D, Hahn M (2010). Extended-connectivity fingerprints. J Chem Inf Model.

[31] Brown MHRD, Varma-O’Brien S, Rogers D (2006). Cheminformatics analysis and learning in a data pipelining environment. Mol Drivers.

[32] Baldi P, Benz RW, Hirschberg D, Swamidass SJ (2007). Lossless compression of chemical fingerprints using integer entropy codes improves storage and retrieval. J Chem Inf Model.

[33] Tanimoto TT. IBM Internal Report 17th (November 1957)

[34] Hert J, Willett P, Wilton DJ, Acklin P, Azzaoui K, Jacoby E, Schuffenhauer A (2004). A. comparison of fingerprint-based methods for virtual screening using multiple bioactive reference structures. J Chem Inf Model.

[35] Hert J, Willett P, Wilton DJ, Acklin P, Azzaoui K, Jacoby E, Schuffenhauer A (2005). Enhancing the effectiveness of similarity-based virtual screening using nearest-neighbor information. J Med Chem.

[36] Nasr RJ, Swamidass SJ, Baldi PF (2009). Large scale study of multiple-molecule queries. J Cheminf.

[37] Geppert H, Horváth T, Gärtner T, Wrobel S, Bajorath J (2008). Support-vector-machine-based ranking significantly improves the effectiveness of similarity searching using 2d fingerprints and multiple reference compounds. J Chem Inf Model.

[38] Mahé P, Ralaivola L, Stoven V, Vert J-P (2006). The pharmacophore kernel for virtual screening with support vector machines. J Chem Inf Model.

[39] Swamidass SJ, Chen J, Bruand J, Phung P, Ralaivola L, Baldi P (2005). Kernels for small molecules and the prediction of mutagenicity, toxicity, and anti-cancer activity. Bioinformatics.

[40] Collobert R, Bengio S (2001). Svmtorch: support vector machines for large-scale regression problems. J Mach Learn Res.

[41] Breiman L (2001). Random forests. Mach Learn.

[42] Palmer DS, O’Boyle NM, Glen RC, Mitchell JB (2007). Random forest models to predict aqueous solubility. J Chem Inf Model.

[43] Zhang Q-Y, Aires-de-Sousa J (2007). Random forest prediction of mutagenicity from empirical physicochemical descriptors. J Chem Inf Model.

[44] Harvey AL (2008). Natural products in drug discovery. Drug Discov Today.

[45] Svetnik V, Liaw A, Tong C, Culberson JC, Sheridan RP, Feuston BP (2003). Random forest: a classification and regression tool for compound classification and qsar modeling. J Chem Inf Model.

[46] Scikit-Learn (2013)

[47] Baldi P, Brunak S (2001). Bioinformatics: the machine learning approach.

[48] Dybowski R, Roberts SJ (2001) Confidence intervals and prediction intervals for feed-forward neural networks. Clin Appl Artif Neural Netw:298–326

[49] Bradley AP (1997). The use of the area under the ROC curve in the evaluation of machine learning algorithms. Pattern Recogn.

[50] Parker CN (2005). Mcmaster university data-mining and docking competition computational models on the catwalk. J Biomol Screen.

[51] Keiser MJ, Roth BL, Armbruster BN, Ernsberger P, Irwin JJ, Shoichet BK (2007). Relating protein pharmacology by ligand chemistry. Nat Biotechnol.

[52] Wale N, Karypis G (2009). Target fishing for chemical compounds using target-ligand activity data and ranking based methods. J Chem Inf Model.

[53] Zaretzki J, Matlock M, Swamidass SJ (2013). Xenosite: accurately predicting cyp-mediated sites of metabolism with neural networks. J Chem Inf Model.

[54] Hinselmann G, Rosenbaum L, Jahn A, Fechner N, Ostermann C, Zell A (2011). Large-scale learning of structure- activity relationships using a linear support vector machine and problem-specific metrics. J Chem Inf Model.

[55] Plewczynski D, von Grotthuss M, Spieser H, Stephane A, Rychewski L, Wyrwicz LS, Ginalski K, Koch U (2007). Target specific compound identification using a support vector machine. Comb Chem High Throughput Screen.

[56] Seifert M, Kraus J, Kramer B (2007). Virtual high-throughput screening of molecular databases. Curr Opin Drug Discov Dev.

[57] Lusci A, Pollastri G, Baldi P (2013). Deep architectures and deep learning in chemoinformatics: the prediction of aqueous solubility for drug-like molecules. J Chem Inf Model.

