# Supplementary material for: Accurate and efficient target prediction using a potency-sensitive influence-relevance voter
Source: J Cheminform. 2015 Dec 29;7:63. doi: 10.1186/s13321-015-0110-6 (PMC4696267; doi:10.1186/s13321-015-0110-6)
Supplement: Supplementary file 2 — 10.1186/s13321-015-0110-6 Additional tables and figures. [file 13321_2015_110_MOESM2_ESM.pdf]

Supplementary Tables and Figures

Alessandro Lusci*, Michael Browning, David Fooshee, S. Joshua Swamidass, and Pierre Baldi*

E-mail: alusci@ics.uci.edu; pfbaldi@ics.uci.edu

* To whom correspondence should be addressed

Cross-Validation Experiment

Table S1: **Average Enrichment performance in the cross-validation experiment on the ChEMBL dataset.** Best results within each group are in bold.

Cutoff = 1μM									
Enrichment	MaxSim	MeanSim	11NN	31NN	51NN	IRV	PS-IRV	SVM	RF
5 %	8	9	8	8	8	**10**	**10**	**10**	**10**
10 %	18	18	16	17	16	**20**	**20**	**20**	19
20 %	34	34	31	33	31	**38**	**38**	37	36
30 %	49	47	45	47	45	**54**	**54**	52	52
Cutoff = 5μM									
5 %	7	7	7	7	7	**8**	**8**	**8**	**8**
10 %	14	15	14	14	14	**16**	**16**	**16**	**16**
20 %	29	29	28	29	27	**32**	**32**	31	31
30 %	42	41	41	42	40	**46**	**46**	45	44
Cutoff = 10μM									
5 %	**7**	**7**	**7**	**7**	6	**7**	**7**	**7**	**7**
10 %	14	14	14	14	14	**15**	**15**	**15**	**15**
20 %	28	27	27	27	26	**30**	**30**	**30**	29
30 %	40	40	40	40	39	**44**	**44**	43	43

Table S2: **Average Enrichment performances in the cross-validation experiment on the external validation dataset.** Models are trained on the ChEMBL dataset. Best results are in bold.

Enrichment	PS-IRV	SVM	RF
Cutoff = 1μM			
5 %	**8**	7	7
10 %	**16**	**16**	**16**
20 %	**30**	**30**	29
30 %	**42**	**42**	41
Cutoff = 5μM			
5 %	**6**	**6**	**6**
10 %	**13**	**13**	**13**
20 %	**27**	26	**27**
30 %	**37**	**37**	**37**
Cutoff = 10μM			
5 %	**6**	**6**	**6**
10 %	**12**	**12**	**12**
20 %	**26**	24	25
30 %	**36**	**36**	**36**

Table S3: **31 and 51 nearest neighbor AUC performances in the fold cross-validation experiment on the ChEMBL Dataset.** Each section of the table shows the average performance for datasets with different sizes.

Cutoff	31NN	51NN
All Datasets		
1 μM	0.79	0.74
5 μM	0.78	0.73
10 μM	0.78	0.72
Datasets with less than 100 molecules		
1 μM	0.68	0.54
5 μM	0.67	0.53
10 μM	0.66	0.53
Datasets with greater than 100 molecules		
1 μM	0.81	0.78
5 μM	0.81	0.78
10 μM	0.81	0.78
Datasets with greater than 200 molecules		
1 μM	0.83	0.80
5 μM	0.84	0.81
10 μM	0.84	0.81

Table S4: **AUC performance and standard deviations in the cross-validation experiment on the ChEMBL dataset.** Each section of the table shows the average performance for datasets with different sizes and the corresponding standard deviations. Best results within each group are in bold.

Cutoff	MaxSim	MeanSim	11NN	IRV	PS-IRV	SVM	RF
All Datasets							
1 μM	0.79	0.76	0.81	**0.86**	**0.86**	0.84	0.84
5 μM	0.76	0.74	0.82	0.84	**0.85**	0.84	0.82
10 μM	0.75	0.73	0.81	0.84	**0.85**	0.84	0.82
Standard deviations							
1 μM	0.10	0.11	0.09	0.10	0.11	0.09	0.10
5 μM	0.11	0.13	0.11	0.13	0.13	0.10	0.13
10 μM	0.12	0.13	0.11	0.13	0.13	0.11	0.13
Datasets with greater than 100 molecules							
1 μM	0.80	0.76	0.73	0.87	**0.88**	0.85	0.85
5 μM	0.77	0.74	0.84	0.87	**0.88**	0.86	0.84
10 μM	0.77	0.73	0.84	0.87	**0.88**	0.86	0.86
Standard deviations							
1 μM	0.08	0.11	0.07	0.08	0.08	0.07	0.08
5 μM	0.10	0.12	0.09	0.09	0.09	0.08	0.10
10 μM	0.12	0.13	0.11	0.13	0.13	0.11	0.13
Datasets with greater than 200 molecules							
1 μM	0.81	0.75	0.84	0.89	**0.89**	0.86	0.86
5 μM	0.78	0.74	0.86	0.89	**0.90**	0.87	0.86
10 μM	0.77	0.73	0.86	0.88	**0.90**	0.87	0.85
Standard deviations							
1 μM	0.07	0.10	0.06	0.05	0.05	0.06	0.06
5 μM	0.09	0.11	0.07	0.06	0.07	0.06	0.07
10 μM	0.09	0.11	0.07	0.08	0.07	0.06	0.08

Table S4: (continued)

Cutoff	MaxSim	MeanSim	11NN	IRV	PS-IRV	SVM	RF
Datasets with greater than 300 molecules							
1 μM	0.81	0.74	0.85	0.89	**0.90**	0.87	0.87
5 μM	0.78	0.73	0.86	0.89	**0.90**	0.88	0.86
10 μM	0.77	0.73	0.86	0.89	**0.90**	0.88	0.86
Standard deviations							
1 μM	0.07	0.09	0.05	0.04	0.04	0.05	0.05
5 μM	0.08	0.10	0.06	0.05	0.06	0.05	0.06
10 μM	0.08	0.11	0.06	0.07	0.06	0.06	0.08
Datasets with greater than 400 molecules							
1 μM	0.81	0.74	0.85	0.89	**0.90**	0.87	0.87
5 μM	0.78	0.73	0.86	0.90	**0.91**	0.88	0.87
10 μM	0.78	0.72	0.86	0.89	**0.91**	0.88	0.86
Standard deviations							
1 μM	0.06	0.08	0.04	0.04	0.04	0.05	0.05
5 μM	0.07	0.09	0.05	0.04	0.05	0.05	0.05
10 μM	0.07	0.09	0.06	0.05	0.06	0.05	0.06

Table S5: **Paired t-test corresponding to the cross-validation experiment on the ChEMBL dataset.** Here we test the statistical significance of the differences between the mean values reported in Table S4. PS-IRV is compared with the other machine learning methods. P-values smaller than 0.05 imply statistical relevance within a 95 % confidence interval.

cutoff	SVM	RF	IRV
All Datasets			
1 μM	< 0.005	< 0.005	-
5 μM	< 0.005	< 0.005	< 0.005
10 μM	< 0.005	< 0.005	< 0.005
Datasets with greater than 100			
1 μM	< 0.005	< 0.005	-
5 μM	< 0.005	< 0.005	< 0.005
10 μM	< 0.005	< 0.005	< 0.005
Datasets with greater than 200			
1 μM	< 0.005	< 0.005	< 0.005
5 μM	< 0.005	< 0.005	< 0.005
10 μM	< 0.005	< 0.005	< 0.005
Datasets with greater than 300			
1 μM	< 0.005	< 0.005	< 0.005
5 μM	< 0.005	< 0.005	< 0.005
10 μM	< 0.005	< 0.005	< 0.005
Datasets with greater than 400			
1 μM	< 0.005	< 0.005	< 0.005
5 μM	< 0.005	< 0.005	< 0.005
10 μM	< 0.005	< 0.005	< 0.005

Table S6: **AUC performance and standard deviations in the cross-validation experiment on the external validation dataset.** Models are trained on the ChEMBL dataset. Each section of the table shows the average performance for datasets with different sizes. Best results within each group are in bold.

Cutoff	PS-IRV	SVM	RF
All Datasets			
1 μM	**0.70**	0.69	0.68
5 μM	**0.69**	0.67	0.67
10 μM	**0.69**	0.66	0.67
Standard Deviations			
1 μM	0.18	0.19	0.18
5 μM	0.19	0.20	0.18
10 μM	0.19	0.20	0.18
Molecules with greater that 100 molecules			
1 μM	**0.71**	0.70	0.70
5 μM	**0.70**	0.68	0.69
10 μM	**0.70**	0.67	0.67
Standard Deviations			
1 μM	0.16	0.17	0.16
5 μM	0.17	0.19	0.17
10 μM	0.18	0.20	0.17
Molecules with greater that 200 molecules			
1 μM	**0.72**	**0.72**	0.71
5 μM	**0.71**	0.69	0.70
10 μM	**0.70**	0.68	0.68
Standard Deviations			
1 μM	0.16	0.16	0.16
5 μM	0.17	0.17	0.17
10 μM	0.17	0.19	0.17

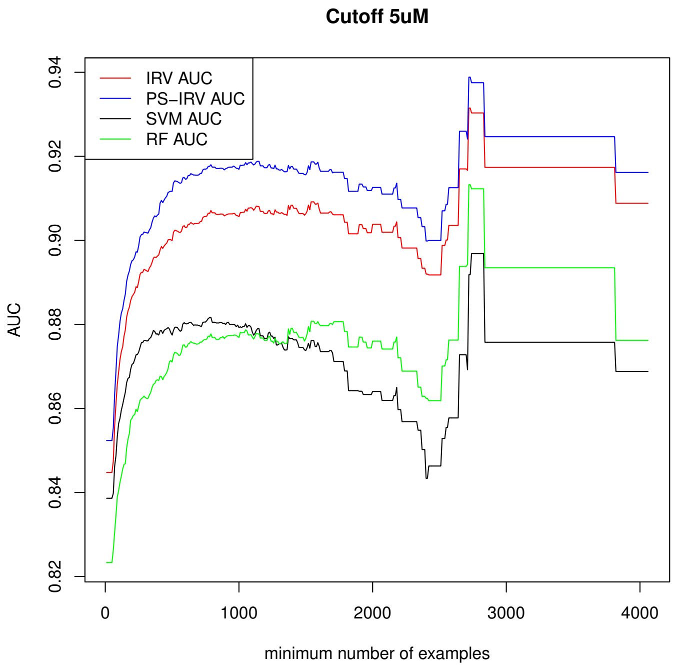

(a) Cutoff = 5 μM

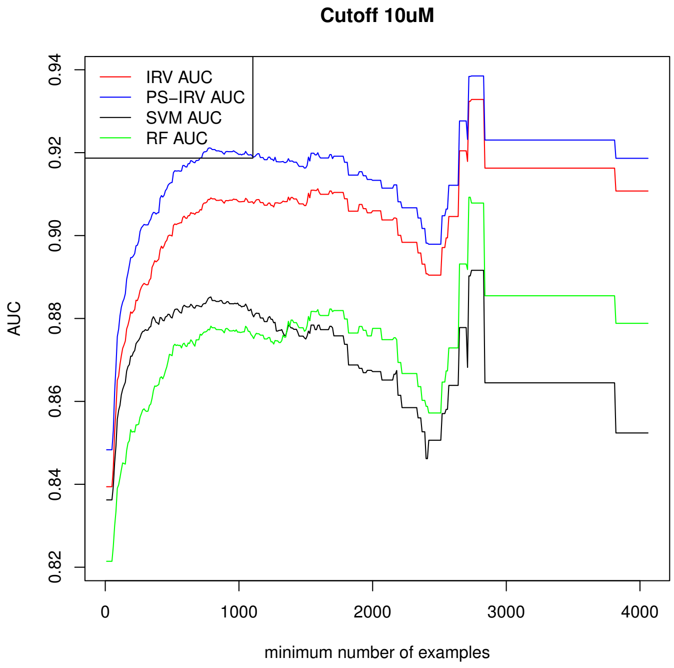

(b) Cutoff = 10 μM

Figure S1: **Cross-validation experiment: AUC scores as dataset size grows**. The average AUC (y-axis) is plotted as a function of the minimum number of training molecules on the x-axis. Model performance (AUC) increases as more datasets with fewer examples are excluded.

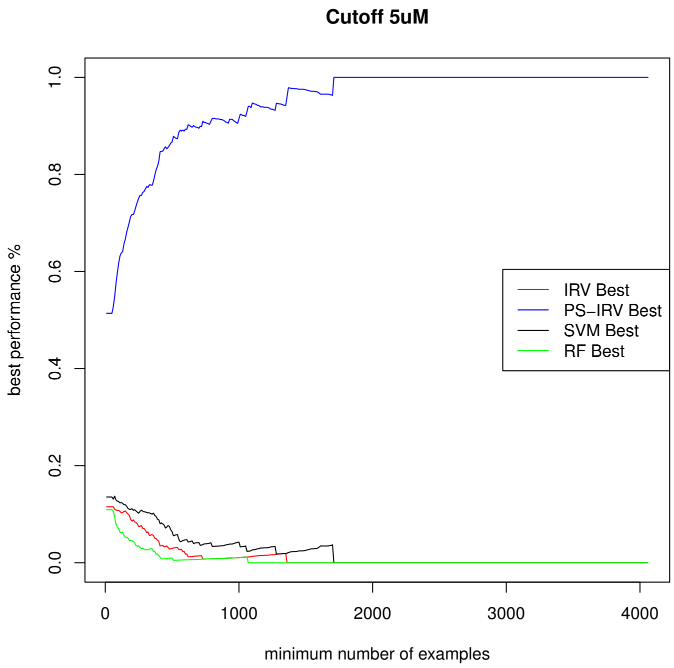

(a) Cutoff = 5 μM

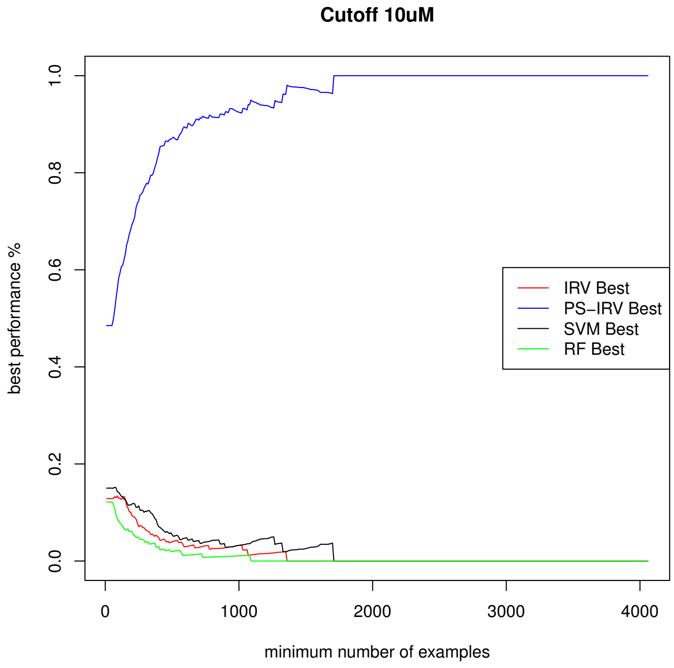

(b) Cutoff = 10 μM

Figure S2: **Cross-validation experiment: best performing models as dataset size grows**. The fraction of times each model achieves the best performance for a dataset is potted on the vertical axis, excluding datasets containing a number of molecules smaller than a specified size. PS-IRV is more consistently the best performer as more of the smaller datasets are excluded.

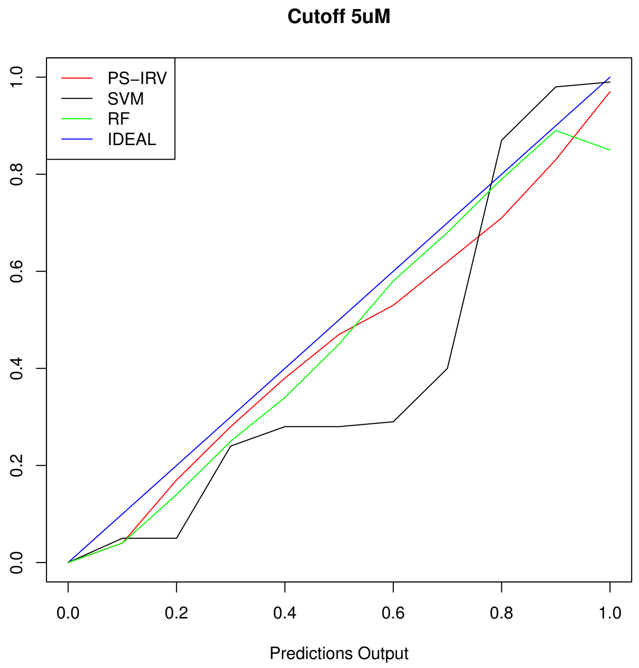

(a) Cutoff = 5 μM

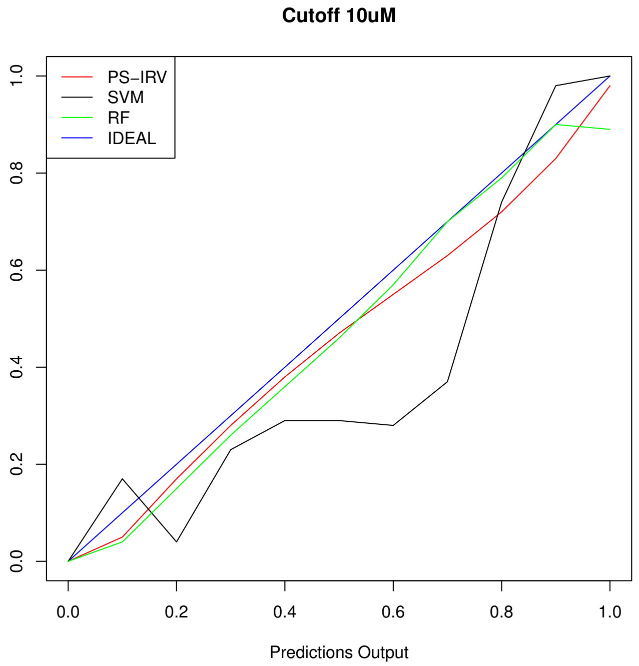

(b) Cutoff = 10 μM

Figure S3: **Probabilistic predictions**. This reliability diagram plot the percentage of positive molecules (y-axis) in the respective bins of molecules with similar prediction values (x-axis). The data is collected from the outputs of the target-prediction models with AUC greater than 0.90. The PS-IRV and RF both produce lines that closely follow the y=x line, indicating that their output can be interpreted as a probability.

Table S7: **Paired t-test corresponding to the cross-validation experiment on the external validation dataset.** Models were trained on the ChEMBL dataset. Here we test the statistical significance of the differences between the mean values reported in Table S6. PS-IRV is compared with the other machine learning methods. P-values smaller than 0.005 imply statistical relevance within a 95 % confidence interval.

Cutoff	SVM	RF
All Datasets		
1 μM	0.33	< 0.005
5 μM	< 0.005	< 0.005
10 μM	< 0.005	< 0.005
Datasets with greater than 100		
1 μM	0.01	0.003
5 μM	< 0.005	< 0.005
10 μM	< 0.005	< 0.005
Datasets with greater than 200		
1 μM	0.04	0.04
5 μM	0.003	0.002
10 μM	0.002	< 0.005

Simulated Target-Prediction

Table S8: **Average Enrichment performances in the simulated target-prediction experiment.** Models are trained using the 10-fold cross-validation protocol and tested on the corresponding test set augmented with 9000 random negative ChEMBL molecules. Best results are in bold.

Enrichment	PS-IRV	SVM	RF
Cutoff = 1μM			
5 %	69	**73**	61
10 %	74	**76**	68
20 %	**81**	79	76
30 %	**86**	81	80
Cutoff = 10μM			
5 %	62	**73**	52
10 %	68	**77**	62
20 %	75	**80**	70
30 %	80	**82**	76
Cutoff = 10μM			
5 %	59	**73**	50
10 %	65	**77**	59
20 %	73	**80**	69
30 %	78	**83**	75

Table S9: **AUC performance and standard deviations in the simulated target-prediction experiment.** Models are trained using the 10-fold cross-validation protocol and tested on the corresponding test set augmented with 9000 random negative ChEMBL molecules. Each section of the table shows the average performance for datasets with different sizes and the corresponding standard deviations. Best results are in bold.

Cutoff	PS-IRV	SVM	RF
All Datasets			
1 μM	0.**88**	0.84	0.84
5 μM	0.84	**0.85**	0.80
10 μM	0.83	**0.85**	0.79
Standard deviations			
1 μM	0.10	0.14	0.18
5 μM	0.12	0.14	0.19
10 μM	0.12	0.14	0.20
Datasets with greater than 100 molecules			
1 μM	**0.92**	0.86	0.87
5 μM	**0.88**	**0.88**	0.84
10 μM	0.87	**0.88**	0.83
Standard deviations			
1 μM	0.06	0.11	0.13
5 μM	0.08	0.11	0.15
10 μM	0.09	0.10	0.15
Datasets with greater than 200 molecules			
1 μM	**0.92**	0.87	0.88
5 μM	**0.90**	0.89	0.86
10 μM	0.89	**0.90**	0.85
Standard deviations			
1 μM	0.05	0.10	0.11
5 μM	0.07	0.10	0.12
10 μM	0.08	0.09	0.13

Table S9: (continued)

Cutoff	PS-IRV	SVM	RF
Datasets with greater than 300 molecules			
1 μM	**0.93**	0.88	0.89
5 μM	**0.91**	0.90	0.87
10 μM	**0.90**	**0.90**	0.86
Standard deviations			
1 μM	0.04	0.09	0.10
5 μM	0.07	0.08	0.11
10 μM	0.07	0.08	0.13
Datasets with greater than 400 molecules			
1 μM	**0.93**	0.88	0.90
5 μM	**0.92**	0.90	0.89
10 μM	**0.91**	**0.91**	0.87
Standard deviations			
1 μM	0.04	0.09	0.09
5 μM	0.05	0.07	0.09
10 μM	0.06	0.07	0.11
Datasets with greater than 500 molecules			
1 μM	**0.93**	0.89	0.90
5 μM	**0.92**	0.90	0.89
10 μM	**0.92**	0.91	0.88
Standard deviations			
1 μM	0.04	0.08	0.09
5 μM	0.05	0.08	0.09
10 μM	0.06	0.07	0.09

Table S10: **Paired t-test corresponding to the simulated target-prediction experiment**. Here we test the statistical significance of the differences between the mean values reported in Table S9. PS-IRV is compared with SVM and RF. P-values smaller than 0.05 imply statistical relevance within a 95 % confidence interval.

Cutoff	SVM	RF
All Datasets		
1 μM	< 0.005	< 0.005
5 μM	0.036	< 0.005
10 μM	< 0.005	< 0.005
Datasets with greater than 100		
1 μM	< 0.005	< 0.005
5 μM	0.048	< 0.005
10 μM	< 0.005	< 0.005
Datasets with greater than 200		
1 μM	< 0.005	< 0.005
5 μM	< 0.005	< 0.005
10 μM	0.26	< 0.005
Datasets with greater than 300		
1 μM	< 0.005	< 0.005
5 μM	< 0.005	< 0.005
10 μM	0.78	< 0.005
Datasets with greater than 400		
1 μM	< 0.005	< 0.005
5 μM	< 0.005	< 0.005
10 μM	0.018	< 0.005
Datasets with greater than 500		
1 μM	< 0.005	< 0.005
5 μM	< 0.005	< 0.005
10 μM	< 0.005	< 0.005

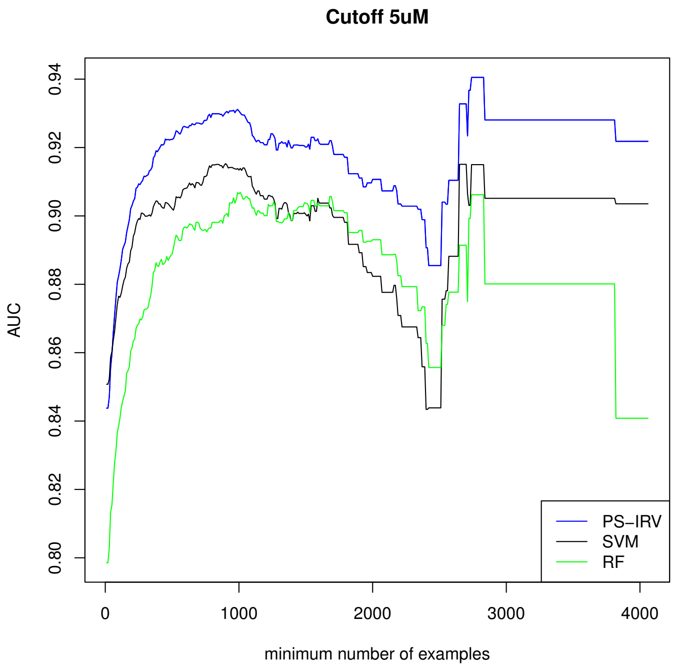

(a) Cutoff = 5 μM

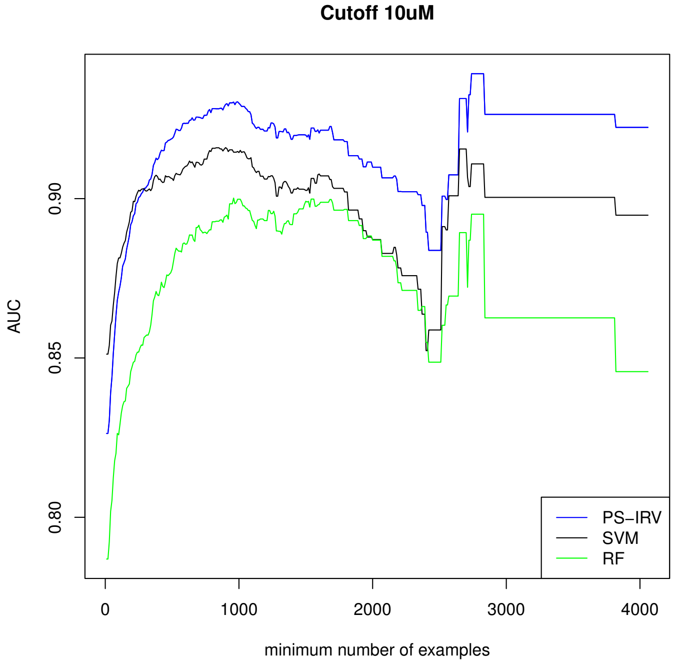

(b) Cutoff = 10 μM

Figure S4: **Simulated target-prediction experiment: AUC scores as dataset size grows**. The average AUC (y-axis) is plotted as a function of the minimum number of training molecules on the x-axis. Each methods ability to separate known actives from a background set of 9000 random ChEMBL molecules, assumed to be inactive, is measured. Training sets are not augmented.

Training With Random Negatives

Table S11: **Enrichment performances in the simulated target-prediction experiment including external validation molecules.** Models are trained using 10-fold cross-validation and tested on the external validation set. Training and test sets are augmented with 1000 and 9000 random negative molecules respectively. Each section of the table show mean and median enrichment values. Best results are in bold.

Enrichment	PS-IRV	SVM	RF
Cutoff 1μ			
5%	**70**	65	66
10%	**76**	72	71
20%	**83**	80	76
30%	**86**	84	78
Median			
5%	**80**	74	77
10%	**90**	84	85
20%	**93**	90	90
30%	**97**	96	92
Cutoff 5μM			
5%	**65**	62	63
10%	**72**	70	69
20%	**78**	77	74
30%	**82**	**82**	76
Median			
5%	**73**	68	71
10%	**84**	81	81
20%	**91**	90	87
30%	**95**	**95**	92

Table S11: (continued)

Enrichment	PS-IRV	SVM	RF
Cutoff 10μM			
5%	**64**	61	62
10%	**71**	69	68
20%	**77**	**77**	73
30%	81	**82**	76
Median			
5%	69	66	**71**
10%	**82**	80	78
20%	**90**	**90**	85
30%	95	**96**	88

Table S12: **AUC performance and standard deviations in the simulated target-prediction experiment when training with random negatives.** Models are trained using the 10-fold cross-validation protocol and tested on the corresponding test set augmented with 9000 random negative ChEMBL molecules. 1000 random inactive molecules are added to the training set. Each section of the table shows the AUC average performance for datasets with different sizes and the corresponding standard deviations. Best results are in bold.

Cutoff	PS-IRV	SVM	RF
All Datasets			
1 μM	**0.98**	**0.98**	**0.98**
5 μM	**0.98**	**0.98**	**0.98**
10 μM	0.97	**0.98**	**0.98**
Standard deviations			
1 μM	0.02	0.02	0.04
5 μM	0.03	0.03	0.03
10 μM	0.04	0.03	0.03
Datasets with greater than 100 molecules			
1 μM	**0.98**	0.97	0.97
5 μM	**0.98**	0.97	**0.98**
10 μM	0.97	0.97	**0.98**
Standard deviations			
1 μM	0.02	0.03	0.03
5 μM	0.03	0.03	0.03
10 μM	0.03	0.03	0.03

Table S12: (continued)

Cutoff	PS-IRV	SVM	RF
Datasets with greater than 200 molecules			
1 μM	**0.98**	0.97	**0.98**
5 μM	**0.98**	0.97	**0.98**
10 μM	**0.98**	0.97	**0.98**
Standard deviations			
1 μM	0.02	0.03	0.03
5 μM	0.03	0.03	0.02
10 μM	0.02	0.03	0.02

Table S13: **Paired t-test corresponding to the simulated target-prediction experiment when training with random negatives**. 1000 random negative molecules are added to the training set. Here we test the statistical significance of the differences between the mean values reported in Table S12. PS-IRV is compared with SVM and RF. P-values smaller than 0.05 imply statistical relevance within a 95 % confidence interval.

Cutoff	SVM	RF
All Datasets		
1 μM	-	-
5 μM	-	-
10 μM	-	< 0.005
Datasets with greater than 100		
1 μM	< 0.005	-
5 μM	< 0.005	-
10 μM	-	-
Datasets with greater than 200		
1 μM	< 0.005	-
5 μM	< 0.005	-
10 μM	< 0.005	-

Table S14: **AUC performance and standard deviations in the simulated target-prediction experiment including external validation molecules.** Models are trained using 10-fold cross-validation and tested on the external validation set. Training and test sets are augmented with 1000 and 9000 random negative molecules respectively. Each section shows the average performances for datasets with different sizes and the corresponding standard deviations. Best results are in bold.

Cutoff	PS-IRV	SVM	RF
All Datasets			
1 μM	**0.90**	0.88	0.87
5 μM	**0.87**	0.86	0.84
10 μM	**0.86**	**0.86**	0.84
Standard deviations			
1 μM	0.14	0.15	0.17
5 μM	0.17	0.17	0.18
10 μM	0.17	0.18	0.18
Datasets with greater than 100 molecules			
1 μM	**0.91**	0.89	0.87
5 μM	**0.89**	**0.89**	0.87
10 μM	**0.88**	**0.88**	0.86
Standard deviations			
1 μM	0.11	0.13	0.14
5 μM	0.14	0.13	0.14
10 μM	0.14	0.14	0.14
Datasets with greater than 200 molecules			
1 μM	**0.91**	0.89	0.88
5 μM	**0.90**	**0.90**	0.88
10 μM	**0.90**	**0.90**	0.88
Standard deviations			
1 μM	0.11	0.13	0.14
5 μM	0.14	0.13	0.14
10 μM	0.14	0.14	0.14

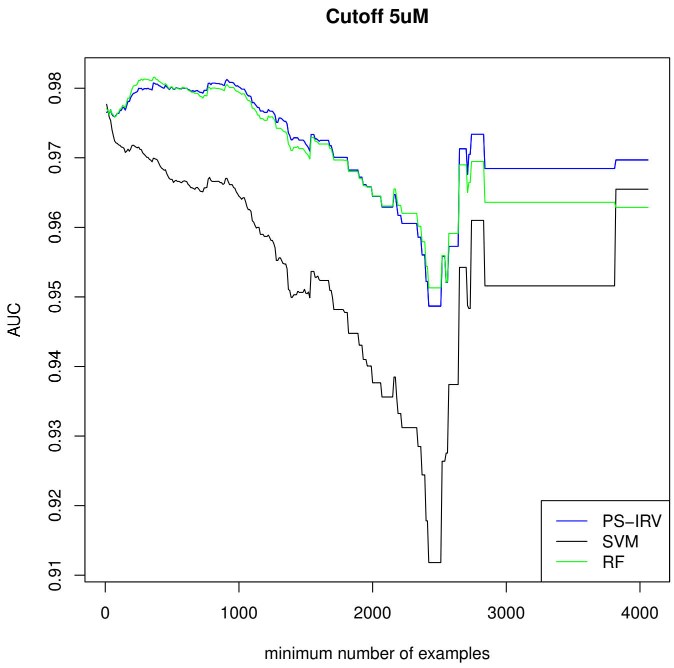

(a) Cutoff = 5 μM

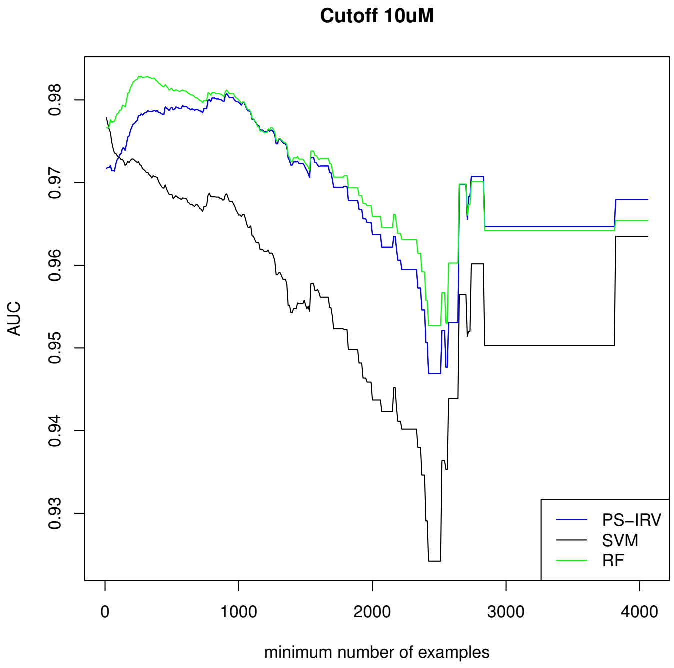

(b) Cutoff = 10 μM

Figure S5: **Simulated target-prediction experiment when training with random negatives: AUC scores as dataset size grows.**The average AUC (y-axis) is plotted as a function of the minimum number of training molecules on the x-axis. Each methods ability to separate known actives from a background set of 9000 random ChEMBL molecules, assumed to be inactive, is measured. 1000 random negative molecules are added to the original training set. The extended training sets result in significant performance improvements.

Table S15: textbfPaired t-test corresponding to the simulated target-prediction experiment including the external validation data. Here we test the statistical significance of the differences between the mean values reported in Table S14. PS-IRV is compared with SVM and RF. P-values smaller than 0.05 imply statistical relevance within a 95 % confidence interval.

Cutoff	SVM	RF
All Datasets		
1 μM	< 0.005	< 0.005
5 μM	0.11	< 0.005
10 μM	-	< 0.005
Datasets with greater than 100		
1 μM	< 0.005	< 0.005
5 μM	-	< 0.005
10 μM	-	< 0.005
Datasets with greater than 200		
1 μM	< 0.005	< 0.005
5 μM	-	< 0.005
10 μM	-	< 0.005
